# The Primate Community of Cachoeira (Brazilian Amazonia): A Model to Decipher Ecological Partitioning among Extinct Species

**DOI:** 10.1371/journal.pone.0027392

**Published:** 2011-11-04

**Authors:** Anusha Ramdarshan, Thomas Alloing-Séguier, Gildas Merceron, Laurent Marivaux

**Affiliations:** 1 Laboratoire de Paléontologie, Institut des Sciences de l'Evolution de Montpellier (ISE-M, UMR-CNRS 5554), Cc 064, Université Montpellier 2, Montpellier, France; 2 Laboratoire de Géologie de Lyon: Terre, Planètes, Environnement, UMR 5276 (CNRS, ENS, Université Lyon 1), Campus de la Doua, Villeurbanne, France; Institut de Biologia Evolutiva - Universitat Pompeu Fabra, Spain

## Abstract

Dental microwear analysis is conducted on a community of platyrrhine primates from South America. This analysis focuses on the primate community of Cachoeira Porteira (Para, Brazil), in which seven sympatric species occur: *Alouatta seniculus*, *Ateles paniscus*, *Cebus apella*, *Chiropotes satanas*, *Pithecia Pithecia*, *Saguinus midas*, and *Saimiri sciureus*. Shearing quotients are also calculated for each taxon of this primate community. Dental microwear results indicate significant differences between taxa, but are somewhat insufficient when it comes to discriminating between ecologically similar taxa. The primates of Cachoeira Porteira all incorporate a certain amount of fruit in their diet, entailing a definite amount of inter-specific competition as they must share food resources. *Alouatta* is the most folivorous taxon of this community, which is corroborated by dental microwear analysis. *Ateles*, although of a similar size to *Alouatta*, limits inter-specific competition by incorporating more fruit in its diet. *Cebus* has a very diverse omnivorous diet, which is highlighted in this study, as it compares to both fruit and leaf eating taxa. In some cases, microwear results need to be supplemented by other methods. For example, dental microwear seems insufficient to distinguish between *Pithecia* and *Chiropotes*, which eat foods with similar physical properties. However, other methods (i.e. shearing quotients and body mass) provide enough complimentary information to be able to highlight differences between the two taxa. On the other hand, dental microwear can highlight differences between primates which have similar diets, such as *Saimiri* and *Saguinus*. In this case, differences could be due to other exogenous factors.

## Introduction

Platyrrhines, or South American monkeys, are a group of diversified primates. From the leaf eating large bodied howler monkeys (*Alouatta*) to the gum gouging tiny marmosets (*Callithrix*), they have colonized a wide array of ecological niches [1]–[3]. Their diets include leaves, fruit, seeds, nuts, insects, gums [2], [4]–[7] and some primates have even been seen to feed on small vertebrates [8]. Most are diurnal, but a few are nocturnal (Owl monkeys [*Aotus*]; [9]) or even in some rare cases, cathemeral (howler monkeys [*Alouatta*]; [10]–[11]). They inhabit or forage in different heights in the forest canopy [12]. These are just a few factors which distinguish different ecological niches seen in South American Monkeys.

Primates are characteristic in that they are not randomly distributed throughout suitable environments, but rather in communities, i.e. taxonomic assemblages of interacting populations [13]–[14]. Living in the same area implies sharing the same resources, which in turn implies some cases of competition between the taxa composing a primate community [15]. Each primate occupies a specific ecological niche characterized by habitat, activity pattern, diet or even foraging behavior. These life history traits are also factors which can limit competition between species. The coexistence of different species in a same environment implicitly implies differences at some ecological level, be it in habitat, diet or in the social organization within the primate community [16]. Diet is of paramount importance in a primate's life [2], [17] and correlates to different aspects of a primate's ecology [18]–[19]. In this study, we focus on this ecological parameter, which will be assessed using three different methods: dental microwear analysis, body mass estimation and shearing quotients.

In the past 30 years, dental microwear analysis has been widely applied to fossil primates and other prehistoric mammals [20]–[29] in order to infer diet. This approach has been proved to be a very useful tool in many paleoecological studies. Ingested food often leaves traces on the surface of dental enamel. This abrasion carries a specific signature depending on the physical nature of the food consumed [21], [23], [30]–[31]. Over the years, microwear has been particularly useful in the dietary reconstruction of extinct primates [20], [23], [29]. This is entirely based on the comparison with modern faunas. However, although microwear studies on extant taxa are widespread, few focus on entire communities [32], where different populations are submitted to the same ecological conditions and above all share the same resources with the same physical properties. In addition, dental morphology is also used to characterize dietary adaptation. While incisors and canines are more involved in obtaining the food, molars are exclusively used to process and reduce it (i.e., mastication). Tooth morphology reflects the physical properties of the food consumed [33]. Studying molar morphology, and specifically shearing crest development, can help to characterize diet. Leaves and insects are difficult to process and require long sharp shearing crests. On the contrary, fruit is generally easier to reduce. Primates which feed on them will generally have teeth exhibiting shorter crests and shallow basins. This observation in extant primates has been quantified [34]–[35] and applied to fossil primates in order to infer diet. Body mass is also an interesting parameter for interpreting diet. Body size directly correlates to many ecological parameters. For example, home range size and even group size tend to increase with body mass [2]. Locomotion also shows a certain correlation with size, terrestrial primates being usually larger than arboreal ones [36]. Body mass also has great influence on diet [35].

These three methods (molar microwear analyses, shearing crest development, and body mass distribution) are applied to one South American primate community, Cachoeira Porteira in the region of Pará in Brazil, where there is a very diverse community of seven species spanning a wide array of ecological adaptations. These methods have previously been used in combination to reconstruct the diet of a fossil primate community from the Eocene of South Asia [37]. The results are then compared to ecological data available for each taxon present at Cachoeira Porteira, with the further aim to consider the implications on fossil species.

## Materials and Methods

### Materials

The material used in this study is housed at the Emilio Goeldi Museum of Pará (MPEG) in Belém (Brazil). It consists of 91 specimens of platyrrhines. All specimens come from the region of Cachoeira Porteira (Para, Brazil) and were collected between 1976 and 1988. These primates lived in a dense primary rainforest environment [38]–[39], with a forest canopy around 50 m above ground. Seven species are present in this primate locality: *Alouatta seniculus*, *Ateles paniscus*, *Cebus apella*, *Chiropotes satanas Chiropotes*, *Pithecia Pithecia Pithecia*, *Saguinus midas midas* and *Saimiri sciureus sciureus.* Their observed diets are very diverse and include leaves, fruit, seeds, nuts, insects and even gums [1], [3], [8], [12], [40]–[46].

### Methods

#### Dental microwear

We made high resolution replicas of individual tooth rows following the protocol laid out by Merceron et al [29]. They were made using a transparent polyester-based resin (Ebalta MG 709-120). Image acquisition then focuses on occlusal surfaces of lower or upper molars. Photos were taken at 100× using an optical stereomicroscope (LEICA M 205 C) connected to a camera (LEICA DFC 420 C). Semi-automatic analysis on images captured through a high-resolution camera and light stereomicroscopy has proven very effective in detecting differences in microwear patterns [24]–[25], [27]–[29], [47]. The resulting grey scale images had a resolution of 3.5 pixels per µm.

Image analysis was conducted with the software ImageJ [48] and the plug-in ObjectJ [49]. For each photo, a standardized square of 100 µm ×100 µm is placed in the centre of the studied crushing facet. During mastication, phase II crushing facets correspond to the surface against which food is reduced [50]–[51]. They are therefore very informative as to dietary habits [31]. Within this standardized square, each microwear scar was quantified as a pit or a scratch, following the definitions of Merceron [29]. Pits have a length to width ratio above ¼ whereas for scratches the same ratio is inferior to ¼. In addition, pits categorized as large if their maximum diameter exceeds 15 µm. Similarly, scratches are considered wide if their width is superior to 15 µm. Seven different variables were analyzed; the number of scratches (Ns), the length of scratches (Ls), the number of pits (Np), the number of wide scratches (Nws), the number of large pits (Nlp), the percentage of pits (Pp), and the total number of scars including scratches and pits (Tot).

The data was then analyzed with the software Statistica (StatSoft) and PAST (PAleontological STatistics, [52]). Statistical analyses were applied to highlight potential inter-group differences in dental microwear patterns. As the conditions for using parametric tests were not fulfilled (i.e., normality), the data was rank transformed before analysis [53]. Individual ANOVAs coupled with Tukey's Honest Significant Difference (HSD) multi-comparison tests were used to pinpoint sources of significant variation.

#### Body mass

Caloric needs and nutritional requirements depend directly on size. Insects are high energy foods, which fulfill the high energy requirements of small bodied primates. Larger animals need fewer calories. Leaves, which are a low in energy but do not involve the same foraging difficulties as insects, can fulfill their requirements. As a result, small bodied primates tend to have a diet based on insects, whereas larger bodied ones (over 500 g) tend to feed on leaves. This natural threshold between the two dietary categories is known as Kay's threshold [35], [54]–[55]. Fruit is high in energy but poor in protein. Fruit eaters will supplement their diet on either insects or leaves according to their size [35]. In this study, we use specimens for which body mass had been directly measured on the captured individuals, and recorded in the database of the Goeldi museum.

#### Shearing quotients

As dental microwear only represents the last few meals of a primate's life, it can be preferable to couple it with other methods. Tooth morphology can also be used to determine diet, as it reflects their mechanical capacities and therefore the physical and structural properties of food [56]. Dental morphological adaptations have been quantified using shearing quotients [34]–[35], [55], [57]–[59]. Shearing quotients (or SQ) correspond to a relative measurement of molar shearing, which strongly correlates to diet. Molar shearing crest are more or less developed according to the nature of a primate's diet. Insects and leaves are composed of chitin and cellulose, respectively, both of which are more resistant to digestion than fruit. Primates that eat them have long, sharp crests suitable for cutting leaves and perforate chitinous exoskeletons. Conversely, frugivores have shorter crests and shallower basins for squashing fruit. The teeth are measured with a microscope fitted with a calibrated reticle (Measuroscope Nikkon 10). For each measurement, the tooth is placed so that the crest being measured is on a horizontal plane. The tooth is then laid out so as to measure the maximum mesio-distal and bucco-lingual lengths of the crown. In this study, we follow the protocol laid out by Anthony and Kay [59] and Kirk and Simons [55]. Only unworn and relatively unworn teeth are used for this method as wear has a direct influence of the length of the crests (a total of 34 specimens).

## Results

### Microwear

Results (Table 1) indicate significant differences in all variables between taxa occuring at Cachoeira Porteira (ANOVA; Table 2). Most of the different diets can therefore be distinguished on the basis of dental microwear analysis.

**Table 1:** Mean and standard deviation for each microwear variable and for each taxon of the Cachoeira primate community.

		Ns		Np		Ls		Nws		Nlp		Pp		Tot	
	N	M	S.e.m	M	S.e.m	M	S.e.m	M	S.e.m	M	S.e.m	M	S.e.m	M	S.e.m
*Alouatta seniculus*	8	25.8	0.7	16.5	0.7	127.3	5.4	0.3	0.2	0.1	0.1	39.0%	3.1%	42.3	1.1
*Ateles paniscus*	9	21.6	0.9	32.9	0.9	141.3	5.3	0.2	0.1	0.8	0.3	60.5%	3.8%	54.4	1.1
*Cebus apella*	22	27.4	1.1	26.7	1.0	64.4	4.8	0.6	0.2	1.1	0.2	49.4%	5.7%	54.1	1.6
*Pithecia pithecia*	5	22.4	0.6	32.6	1.2	77.0	1.8	2.6	0.5	2.4	0.6	61.0%	3.5%	55.0	1.1
*Chiropotes satanas*	17	21.6	0.9	33.6	0.9	71.9	2.9	1.8	0.2	2.9	0.3	59.2%	2.9%	55.3	1.6
*Saguinus midas*	7	27.1	0.7	33.6	1.2	76.5	2.6	0.3	0.2	0.4	0.3	55.3%	2.1%	60.7	1.6
*Saimiri sciureus*	5	16.4	1.3	33.0	1.0	79.9	1.7	0.2	0.2	0.4	0.2	66.9%	4.3%	49.4	1.6

Abbreviations are as follows: N: number of specimens (one specimen corresponds to one individual); Ns: number of scratches; Ls: scratch length; Np: number of pits; Nws: number of wide scratches; Nlp: number of large pits; Pp: percentage of pits; Tot: total number of microwear scars.

**Table 2:** Univariate analyses of variance with ranked data.

ANOVA		S.S.	d.f.	M.S.	*F*	*p*
Ns	Effect	15187.97	6	2531.33	9.8090	<0.05
	Error	17032.03	66	258.06		
Ls	Effect	20162.31	6	3360.39	18.1061	<0.05
	Error	12249.19	66	185.59		
Np	Effect	18601.28	6	3100.21	14.9613	<0.05
	Error	13676.22	66	207.22		
Nws	Effect	14023.67	6	2337.28	11.0717	<0.05
	Error	13932.83	66	211.10		
Nlp	Effect	14948.36	6	2491.39	10.9427	<0.05
	Error	15026.64	66	227.68		
Pp	Effect	24935.37	6	4155.89	36.7230	<0.05
	Error	7469.13	66	113.17		
Tot	Effect	12374.99	6	2062.50	6.8245	<0.05
	Error	19946.51	66	302.22		

Abbreviations for microwear variables are as in Table 1.

*Alouatta seniculus*, a leaf-eating primate, differs from all the other taxa of this primate community in having fewer pits (Table 1 & 3) and a high number of scratches. *A. seniculus* is also characterized by a lower total number of microwear scars than fruit-eaters such as *Saguinus midas* or *Pithecia pithecia* and than the most omnivorous species *Cebus apella*. Microwear results are in agreement with the highly folivorous for *A. seniculus* in Cachoeira Porteira.

**Table 3:** Tukey′s HSD pairwise comparison test.

	*Alouatta seniculus*	*Ateles paniscus*	*Cebus apella*	*Pithecia Pithecia*	*Saguinus midas*	*Saimiri sciureus*
*Alouatta seniculus*						
*Ateles paniscus*	Np, Pp, Tot					
*Cebus apella*	Np, Ls, Pp, Tot	Ns, Np, Ls, Pp				
*Pithecia pithecia*	Np, Ls, Nws, Nlp, Pp, Tot	Ls, Nws	Np, Nws, Pp			
*Saguinus midas*	Np, Ls, Pp, Tot	Ns, Ls, Pp	Np	Nws, Nlp		
*Saimiri sciureus*	Ns, Np, Pp	Ls	Ns, Np, Ls, Pp	Nws, Nlp, Pp	Ns, Pp, Tot	
*Chiropotes satanas*	Ns, Np, Ls, Nws, Nlp, Pp, Tot	Ls, Nws, Nlp	Ns, Np, Nws, Nlp, Pp		Ns, Nws, Nlp, Pp	Nws, Nlp

Abbreviations for microwear variables are as in Table 1.

**Figure 1:**
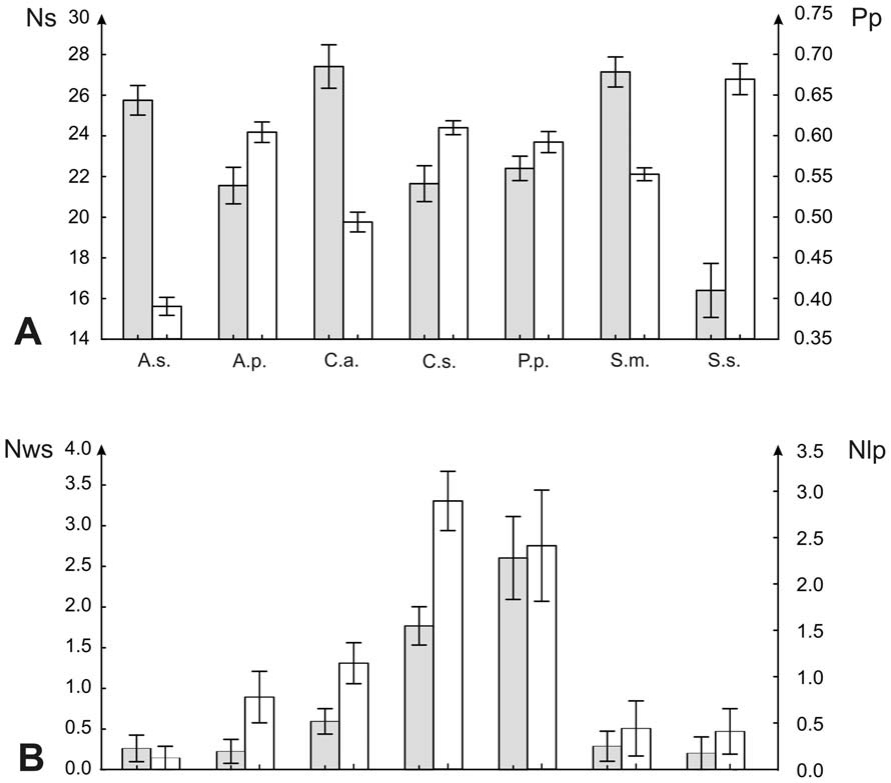
Mean and standard error of the mean for different microwear variables. A, number of scratches (Ns) and pit percentage (Pp); B, number of wide scratches (Nws) and of large pits (Nlp). Abbreviations for taxa are as follows: A.s. *Alouatta seniculus*; A.p. *Ateles paniscus*; C.a. *Cebus apella*; C.s *Chiropotes satanas*; Pp. *Pithecia Pithecia*; S.m. *Saguinus midas*; S.s. *Saimiri sciureus*.

*Chiropotes satanas*, a so called hard object feeder has a lower number of scratches than the leaf eating *A. seniculus* (Table 1,2 &3), relating to the different proportions of leaves in their diet (Figure 1). *C. satanas* also show a higher number of wide scratches and large pits than the soft fruit eating *S. midas* or *S. sciureus*. However, no significant differences were highlighted between the two hard object feeders, *C. satanas* and *P. pithecia*, who shows the same differences with respect to other taxa. Both *C. satanas* and *P. pithecia* show a higher number of pits than the leaf eating *A. seniculus*.

*Ateles paniscus* shows a lower number of scratches and a higher number of pits than *A. seniculus*, which is coherent as it is the more frugivorous of the two. *A. paniscus* differs from the hard object eaters (i.e., *P. pithecia* and *C. satanas*) in having fewer large pits and wide scratches. *A. paniscus* differs from all the other primates from Cachoeira Porteira by a higher scratch length.

*Cebus apella* shows a higher number of scratches than most fruit eaters of the community (Table 1), and a higher number of pits than the leaf eating *A. seniculus*. *Cebus* also has a higher number of wide scratches and large pits *A. seniculus* and soft fruit eaters (*A. paniscus*, *S. midas*). However, these values are also lower than those seen in hard object feeders such as *P. pithecia*.

*Saguinus midas* differs from leaf eaters by having more pits and fewer scratches (Table 1, 2 & 3). *S. midas* also has significantly more pits than the omnivorous *C. apella*. Although *S. midas* and *S. sciureus* have a similar diet, microwear patterns show significant differences. *S. midas* has a significantly higher number of scratches than *S. sciureus*. Similarly to *S. midas*, *S. sciureus* has significantly more pits and fewer scratches than the leaf eating *A. paniscus*.

### Body mass and shearing quotients

**Figure 2:**
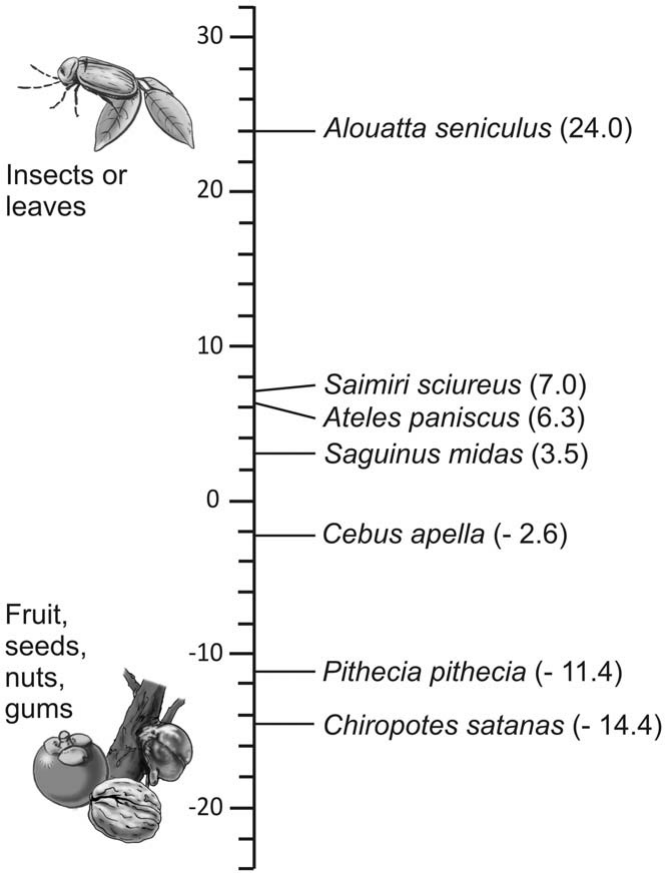
Comparison of the shearing quotients of the different primates of Cachoeira Porteira. Low values indicate a diet based on fruit, gums or even seeds whereas a high SQ points towards a leaf or insect based diet.

All the primates figured in this study are above Kay's threshold of 500g (Table 4a). As such, they are too big to be able to depend exclusively on insects. *A. paniscus* has a higher SQ (indicating a leaf-based diet) than the other primates, which all incorporate fruit in their diet (Table 4b; Figure 2). *A. paniscus*, although being of a similar size to *A. seniculus*, clearly distinguish itself from the latter by a lower SQ, which indicates fruit eating. *S. sciureus* had more developed shearing crests than the similar sized *S. midas*, indicating this taxon should incorporate a larger amount of insects in its diet than *S. midas*. Both *C. satanas* and *P. pithecia* have very negative SQs, with values below those of fruit eaters such as *S. midas* or *A. paniscus*. Such poorly developed shearing crests are indicative of hard object feeding.

**Table 4:** (a) Mean and standard deviation of body mass for each species (Values are in grams).

(a) Body mass estimations (g)			
Taxon	N	Mean	SD
*Alouatta seniculus*	8	5594	1821
*Ateles paniscus*	9	6800	3427
*Cebus apella*	34	2538	1033
*Chiropotes satanas*	23	2546	681
*Pithecia pithecia*	5	1768	430
*Saguinus midas*	7	491	43
*Saimiri sciureus*	5	780	157

(b) Shearing quotients			
Taxon	N	Mean	SD
*Alouatta seniculus*	5	24	1.6
*Ateles paniscus*	5	6.3	1.3
*Cebus apella*	7	−2.6	1.7
*Chiropotes satanas*	7	−14.4	1.8
*Pithecia pithecia*	3	−11.4	1.3
*Saguinus midas*	4	3.5	1.3
*Saimiri sciureus*	3	7	1.5

(b) Shearing quotients for each taxon. Abbreviations are as follows: N: number of specimens; M: mean; SD: standard deviation; SQ: shearing quotient.

## Discussion

Dental microwear analysis has highlighted significant differences between the different primates of this community. Although seasonality is known to affect diet and microwear patterns in extant primates [60], the specimens included here were all collected during the same time period. As the all the taxa of this primate community were sampled during the same seasons, differences in microwear patterns cannot be attributed to seasonality but rather reflect dietary and ecological differences.

**Figure 3:**
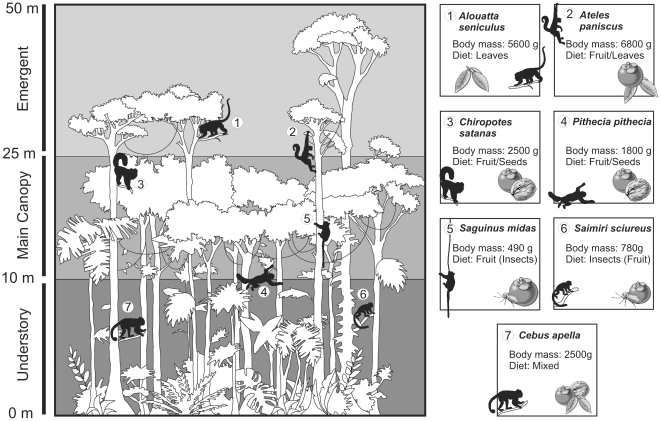
Summary of the ecological data available for the Cachoeira Porteira primate community. Each species of the Cachoeira primate community is represented in its preferred habitat (understory [0–10 m], main canopy [10–25 m] or emergent [25–50 m]). Body mass and diet are also summarized for each taxon. Ecological data taken from the literature: [1], [2], [3], [8], [12], [40], [42]–[46].

The seven species studied here are all diurnal. *Alouatta seniculus* is a leaf eater [1], [42]. This taxon is also the only primate at Cachoeira Porteira to incorporate leaves as the main dietary component. The outcome from our shearing quotient and dental microwear analyses fits well this diet. *A. seniculus* occupies a very different niche to the other primates in this locality (Figure 3). In fact, all of the other primates are fruit eaters, entailing a definite amount of inter-specific competition as they share food resources. Every taxon incorporates a certain amount of fruit in its diet, which in turn entails a certain amount of inter-specific competition. *Ateles* (6800 g) is the only other primate of a similar size to *A. seniculus* (5600 g), pointing towards similar ecological niches. They also forage in the same levels, i.e. the upper levels of the main canopy and emergent of the high forest (Fig. 3), although *Ateles paniscus* seems to be slightly more restricted in distribution [40], [45]. Dental microwear and shearing quotient indicate this taxon has a diet based mainly on fruit. *A. paniscus* does predominantly eat fruit and supplements its diet with leaves [45]. Ecological overlap between *A. paniscus* and *A. seniculus* is thus very limited as they incorporate very different quantities of fruit in their diet.

Dental microwear analysis is efficient in distinguishing different dietary categories. For example, a high number of scratches differentiates leaf-eaters (i.e., *Alouatta seniculus*) from the other fruit-eaters of the primate community. Similarly, a high number of large pits and wide scratches are indicative of hard object feeding (i.e., *Cebus apella* and *Pithecia pithecia*). However, dental microwear fails in some cases to highlight any difference between taxa which are ecologically close. For example, no differences were shown between the two hard object feeders of this primate community (*Pithecia pithecia* and *Chiropotes satanas*). Both taxa feed on fruit and prefer hard object such as seeds and nuts. They are considered as seed predators [3], husking fruit which has a hard pericarp and then eating the softer but much tougher seed contained inside [43]. They have colonized a very specialized ecological niche. Their preferred foods have similar physical properties so it is not surprising that dental microwear patterns are so similar between the two taxa. Although they do have overlapping niches, *P. pithecia* and *C. satanas* do differ ecologically. One notable difference is their choice of fallback food: when fruit becomes scarce, *P. pithecia* eats more flowers whereas *C. satanas* prefers fruits with harder pericarps [12]. However, as microwear only imprints the last days or few weeks of a primate's life [61], these differences are not necessarily recorded by the studied sample. In fact most of the ecological differences between the two taxa would not have an influence on microwear patterns. One difference lies in the type of plant each taxa targets. Although the physical properties of the fruits eaten by *P. pithecia* and *C. satanas* are similar, the chemical properties are not. *P. pithecia* tends to avoid plants with high tannin levels [12]. Again, these differences would not have any incidence on dental microwear patterns. In this case, dental microwear does not seem sufficient in discriminating dietary niches. However, *P. pithecia* and *C. satanas* can be differentiated by their shearing quotient. *Chiropotes* displays less developed molar shearing than *P. pithecia*. Different dental features thought to be “adaptations” might reflect the physical properties of fallback foods rather than preferred foods [62]. Preferred foods will require little specialization [62]-[63]. For example, soft fruit is easily processed. On the other side of the spectrum, fallback foods, such as fruit with a hard pericarp, are going to require a higher degree of specialization because they are less easily processed [63]. Although both values indicate hard object feeding, the differences in SQ seen in *P. pithecia* and *C. satanas* might reflect their differences in fallback foods (i.e. flowers *vs.* fruit with a hard pericarp). Other ecological differences cannot be interpreted from dental microwear or shearing quotients. For example, *P. pithecia* is most often found on lower levels of the forest understory, contrary to *C. satanas*, which prefers higher canopy levels [40]. This vertical stratification implies that these two species do not forage at the same height, thus limiting inter-specific competition. Although dental microwear and shearing quotient are unable to help in this matter, body mass can. It has been linked to locomotor behavior, notably among platyrrhine monkeys [40]. For example, in South American communities, leaping is more common for smaller than for larger species, whereas suspensory behavior is more commonly seen in larger species than in smaller ones [2]. Different locomotor habits provide different types of access to different parts of a forest habitat [64]. Body mass has also been linked to the size of the branches used, which in turn can indicate different forest heights [16]. Estimates for these taxa indicate that *P. pithecia* (1700g) is slightly smaller than *C. satanas* (2500 g), which in this case points towards different forest levels.

*Cebus apella* has a very diverse mixed fruit and leaf diet, including foods as diverse as nuts, seeds, insects, eggs, lizards and even small vertebrates and mammals [8], [41], [46]. This omnivorous diet is highlighted in the dental microwear patterns, as it compares to leaf and fruit eating taxa in terms of number of pits and scratches. However, shearing quotients alone seem insufficient to characterize *C. apella*'s diet, as it only highlights fruit eating. Previous studies [46], [65] have considered *C. apella* as a hard object feeder. Although this taxon does incorporate a certain amount of seeds in its diets, it is not to the same extent as seed predators such as pithecines. This ecological observation is also highlighted by our results. Indeed, *C. apella* does display a high number of pits, but it remains significantly lower that those of the two pithecines (i.e., *P. pithecia* and *C. satanas*).

*Saimiri sciureus* and *Saguinus midas* have similar diets. *S. sciureus* has a diet mainly based on insects with a large secondary component of fruit [40], [44]–[45] whereas *S. midas* has a diet mainly based on fruit and heavily supplemented by insects. This is reflected in their dental microwear patterns. Few significant differences were highlighted in this analysis. The main difference in their microwear patterns lies in the number of scratches. However, differences between the proportions of fruit and insects should not have any physical consequences in the number of scratches, but rather on the number of pits. So the different number of scratches seems difficult to explain. Other exogenous factors may influence microwear. Dust has been proved to influence dental microwear patterns, even among tropical rainforest dwelling primates [66]. It is present even at higher levels of the forest canopy. It is present in all levels of the forest and even at higher levels of the canopy [66]. Foraging at different levels could then have an influence on dental microwear patterns. *S. midas* and *S. sciureus* have a similar diet but a different vertical distribution. *S. midas* prefers the middle and lower levels of the main canopy, whereas *S. sciureus* is more often found in the understory than in higher levels [45]. Foraging at different heights could explain the different number of scratches observed in *S. midas* and *S. sciureus*. Dental microwear analysis does seem pertinent for distinguishing ecological niches in this case. Shearing quotients also differentiates between these two taxa, showing a greater capacity towards fruit eating in *S. midas*, which displays less developed molar shearing than *S. sciureus*.

### Conclusions and implications for fossil primate communities

Diet is of paramount importance in a primate's life, correlating to a wide array of ecological factors. It is therefore a pertinent tool in the characterization of ecological niches. The methods used in this study have indeed allowed us to discriminate between the different diets present among the primates at Cachoeira Porteira. Although dental microwear analysis is very useful for interpreting diet from dental remains, it is sometimes insufficient to describe the full spectrum of a primate's diet. In this study, we were able to differentiate between most of the species present at Cachoeira Porteira, but difficulties arose when comparing primates with similar overlapping ecological niches.

For example, we cannot differentiate between *Pithecia* and *Chiropotes* on the basis of dental microwear patterns alone. Other methods are necessary in order to attain a wider spectrum of information. *Pithecia* and *Chiropotes* can be differentiated using body mass and shearing quotients. These two elements contribute towards the ecological characterization of the primates of Cachoeira Porteira.

The methods used in this study have the advantage to be easily applicable to fossils. Teeth are the most mineralized parts of an animal, and are much more resistant during diagenesis than cartilage or bone. Thus, they constitute a major part of the mammal fossil record. A most important application of dental microwear analysis is the reconstruction of the diet of fossil vertebrates. This method has been applied to various groups with success [20], [22]–[23], [33], [37]. Correct interpretation of microwear patterns relies entirely on the comparison with extant groups. In this study, comparison between the microwear results and ecological data available for each taxon highlighted certain difficulties.

Microwear only imprints the last weeks or even days in the life of the animal (depending on the nature of its diet). Teaford et al. [61] proved that microwear patterns are quickly replaced by whatever imprints the next feeding leaves behind. In some cases, microwear can be erased after only 24 hours. Thus, dental microwear patterns are, at best, a direct record of the last meals before death and not necessarily an indication of the overall diet of the animal. This method can only differentiate on the basis of physical properties, not on differences in chemical composition as that does not have any influence on scarring the enamel. A primate's diet also includes some soft foods (e.g., gums, flowers) that do not leave imprints in the enamel of the teeth [67]. Other exogenous particles have been proved to have an influence on dental microwear patterns. For example, dust can be constituted from silicate particle or exogenous quartz, both of which are hard enough to be able to scratch enamel. Therefore, some of the observable microwear on the dental surfaces could come from such elements, and not reflect the diet in any way. Ungar et al. [66] have shown the presence of particles and dust in the canopy. Be it in an open environment or a tropical rainforest, they accumulate in the primate's potential food and can thus influence the microwear patterns while having nothing to do with the diet itself. This can bring different signals into microwear patterns, which become very difficult to distinguish when ecological data isn't available for comparison.

However, together with other methods for dietary reconstruction, higher resolution can be achieved. For example, shearing quotients and body mass permitted us to differentiate *Pithecia* and *Chiropotes*, where microwear alone couldn't. Other taxa have similar shearing crest development, but different microwear patterns. While dental microwear is a direct recording of the animal's last meals, the shearing quotient takes into account the morphological adaptations of its teeth to suit its diet. Many dental characteristics are both the reflection of a phylogenetic heritage as much as of a function [68]. Thus, the differences in shearing crest length can express phylogenetic history as well as adaptive difference.

Both methods rely on analogies between modern and fossil faunas. This does not take into consideration that fossil species might show different adaptations to those found in modern species. The expression of the adaptations among the different groups of primates has probably changed over time [2]. That said, the same diet can be expressed by different biological adaptations or feeding strategies. Both have disadvantages, hence the interest in using them to supplement one another.
